# Of Fears and Budgets: Strategies of Control in *Vespa velutina* Invasion and Lessons for Best Management Practices

**DOI:** 10.1007/s00267-022-01690-z

**Published:** 2022-07-28

**Authors:** Tamara Pazos, Patricia Álvarez-Figueiró, Jose A. Cortés-Vázquez, María Amalia Jácome, María J. Servia

**Affiliations:** 1 Department of Biology, Faculty of Science, University of A Coruña, UDC, Campus da Zapateira s/n, 15071A Coruña, Spain; 2 Department of Sociology and Communication, Faculty of Sociology, University of A Coruña, UDC, Campus de Elviña s/n, 15071A Coruña, Spain; 3 Department of Mathematics MODES Group, Faculty of Science, CITIC University of A Coruña, UDC, Campus da Zapateira s/n, 15071A Coruña, Spain

**Keywords:** *Vespa velutina*, Nest removal, Emergency management, Economic cost, Administration, Communication

## Abstract

Implementing management practices for the control of invasive species can be a complex task with multiple dimensions, where the identification of stakeholders and drivers of those practices is of paramount importance. The invasive hornet *Vespa velutina* has spread across Europe and Asia from its native range in SE Asia in recent years. A common control method is the removal and destruction of its nests on citizens’ request to call centers. In this paper we have explored the knowledge and main factors that influence the perceptions of the citizens on the species in an invaded municipality in NW Spain, as well as the management practices of the municipal emergency unit responsible for nest removal activities. Our analysis brings out multiple drivers of management practices that derive both from the citizens’ and practitioners’ knowledge, and highlights several points of conflict between both stakeholder groups connected to (1) the degree of service provided to the local population, (2) the risk of allergic reactions as a motive to urge removals, or (3) the quality of information provided by mass media. Our results support the crucial importance of environmental education programs that seek to increase the knowledge of the general public about the threats of invasive species. Such programs might be incorporated to implement and optimize management plans of *V. velutina* by enhancing communication between experts and local population.

## Introduction

Within the realm of biology, debates around invasive alien species (IAS) go from foundational definitions to the availability of evidence of their impact (Davis 2020). Discussions on IAS also abound within the field of environmental management, where there seems to be a growing consensus about the need of considering both the environmental and socio-economic context in making decisions about biological invasions (Larson et al. 2011). The latter is particularly important with regard to the participation and/or involvement of affected communities, both in surveilling the presence and expansion of the species, as well as in invasion control (Marzano et al. 2017). Although the importance of community participation is well established in the literature (e.g., Hester and Cacho 2017), there is still a need to define best management practices in order to influence the outcome of such control programs, which might range from the efficient eradication of the species, to promoting its spread or other unwelcome side-effects.

The case of *Vespa velutina* invasion control is paradigmatic of the need of defining best management practices in community engagement activities in IAS management programs. The invasive Asian hornet *V. velutina* has spread across Europe from its native range in SE Asia in recent years. It was accidentally introduced in France before 2004, and the species is nowadays present in most of Western Europe, where eradication is considered to be no longer possible (Robinet et al. 2017). The species has invaded also important areas in South Korea and Japan, and it has become a notable troublesome IAS (Do et al. 2019). The need of quick actions has led to the implementation of community-participated control practices without the necessary provision of rigorous information to the public, leaving these practices at the mercy of diverse factors, from stakeholder needs to social beliefs, risk perception and fears (Requier et al. 2020).

This paper analyzes management practices of *V. velutina* invasion at a fine scale with the aim of making two contributions, one general and theoretical and another one more applied and specific. Firstly, we will reflect on common pitfalls in the “co-production” of knowledge and control practices within biological invasions. We follow Jasanoff’s (2004) definition of co-production as the necessary implication of the knowledge of both experts and lay citizens in the implementation of environmental management practices. We develop this concept into the field of biological invasions by exploring how, while indispensable, such form of expert/non-expert collaboration at many levels might also hamper control efforts if not mediated by adequate environmental education interventions. This is a particularly pressing issue when budgets are constrained, which tends to be the background context of invasion control (Courtois et al. 2018). In such context, best management practices hinge on better optimal resource allocation–a goal to be reached by forming and training lay citizens in some key principles of invasion biology.

Secondly, we apply this theoretical elaboration into the control and management practices of *V. velutina* invasion. We will analyze the use of nest removal in the management control of *V. velutina* in a municipality of Galicia, a region in NW Spain. We will explore how the problems encountered by this management control program might be connected to the differences in environmental knowledge and motivations between experts and citizens. Our aim is to identify how generally shared, non-scientific perceptions on this species and emotion-driven interactions between public administration and citizens may play an important role in shaping co-produced control activities. We argue that the study of such beliefs and attitudes is a first step to help create more effective and standardized management strategies for invasive species.

### The Biology of *Vespa velutina* Invasion

*Vespa velutina* Lepetier 1836 is a diurnal species of Hymenoptera with an annual biological cycle, which presents its maximum activity during late summer and early autumn. The cycle begins in early spring, with fertilized queens leaving their hibernation refuges to begin the construction of a primary nest. The colony grows at increasing speed as new workers contribute to construction and feeding activities. During the summer the colony may move to a new place, frequently high tree canopies, where they build a bigger nest. These nests can attain 40–50 cm in diameter and support few thousands of individuals during their maximum period of activity (Rome et al. 2015). In autumn males and fertile gynes (i.e., future queens) are produced, and gynes are the only individuals to overwinter while the rest of the colony dies. Nests are not reused in the following season (Monceau et al. 2014).

In contrast with the moderate spread of *V. velutina* in invaded areas in South Korea and Japan due to competition with native species of hornets (Kwon and Choi 2020), its spread in Europe has been very rapid (Robinet et al. 2017). The invasion has been accompanied by important environmental and socio-economic impacts because of its intense predation pressure on honeybees and behavioral disturbances to other insects (Rojas-Nossa and Calviño-Cancela 2020). Also, reports of deaths by stings and other health risks have increased the attention given to this species (Feás 2021). *Vespa velutina* has been cataloged as an invasive alien species of concern in Europe and is considered a high impact species by the European Commission (EU 2014). In Spain, it has been included in the catalog of invasive alien species under the regulation RD 630/2013.

A notable ecological impact derives, paradoxically, from programs intended to control the species. One of the most popular methods among citizens in invaded areas is the use of liquid baited traps in plastic bottles, a simple and cheap method that is highly controversial because of its low selectivity (Lioy et al. 2020; Rojas-Nossa et al. 2018; Rome et al. 2021) and the lack of evidence on its efficiency to limit the distribution of *V. velutina* (Monceau and Thiéry 2017). Although some administrations have promoted trapping programs, most of them have dedicated important resources to nest destruction for controlling the invasion, with important associated economic costs (Barbet-Massin et al. 2020; Choi et al. 2019).

### Co-production of Environmental Knowledge and the Management of *V. velutina*

Biodiversity studies, including those on invasion biology, have benefited from the integration of different knowledge systems in recent decades (Marzano et al. 2017), following a more general trend in what Jasanoff (2004) defines as the co-production of science. Co-production is an analytical term to scrutinize how science, policy, and practice co-evolve. Concepts such as Traditional Environmental Knowledge (TEK) or Local Knowledge have been revised and debated regarding the role they can play in the co-production of science (Berkes et al. 2000; Hill et al. 2020). TEK includes beliefs, values and criteria which guide local practices in relation to nature based on historical interactions and involvements with the surrounding environment (Berkes et al. 2000, Sillitoe 2006). It has been demonstrated that taking this kind of knowledge seriously increases the appeal and support of management activities among local groups (García-Llorente et al. 2011; Shackleton et al. 2019a).

The need for citizen commitment and community engagement is also recognized as a requirement to treat conservation problems such as biological invasions (Shackleton et al. 2019b). This need for involvement is important even in the absence of TEK (for example, among new settlers in rural areas), because local citizens can be a powerful ally by helping with data collection (from passive detection to citizen science), disclosure, active surveillance or simply by changing their behavior towards more environmentally friendly practices.

However, when implementing community engagement activities in pest and disease management programs, one of the most common obstacles is the discrepancy in perceptions that different stakeholders (e.g., local communities, authorities, experts) may have about management strategies or priorities. This often leads to misunderstandings, inefficiency, and even conflict (Shackleton et al. 2019a). As a result, the study of beliefs or attitudes that influence people’s behavior can help gather information for the creation of environmental education campaigns and more effective management of invasive species (Prinbeck et al. 2011).

The management of *V. velutina* invasion in Galicia, NW Spain, is a good example of community engagement, in terms of both co-production of knowledge and control of the invasion. It can help us illustrate the complexity of coproduced management strategies.

In 2014 a protocol was published by the Galician administration with the broad objective of monitoring and controlling *V. velutina*. This protocol included recommended methods for capturing hornets (through the use of baited traps) and removing nests, as well as an overview of the main responsibilities of regional departments. It included also a call center number for nest removal requests that could be used by any citizen, which might be considered a passive surveillance system *sensu* Hester and Cacho (2017). A subsequent update in 2016 incorporated specific decision flow charts for nest removal where the coordination was assumed by the regional administration, but most of the responsibility for management actions and provision of resources lay with municipalities. In 2020 a new management strategy was agreed between the regional and local administrations, and the collaboration of municipal emergency services was requested only in case of urgency or specific needs (Xunta de Galicia 2021). The same call center number is in use at present for nest removal requests by any person in the region.

Nests are removed only upon citizens’ request and it is free of charge, as in other areas (Choi et al. 2019). Citizens dial a call center when they detect a nest and the request is then transferred to the municipal emergency groups, which register the basic data of the removal action in a central database. Therefore, citizens play a paramount role since nest detection and communication of findings rely on them. Because of this, their requests directly influence the work of emergency services, both in terms of their quality and quantity (e.g., generating high peaks of requests). Consequently, human perceptions on this species and interactions among groups with different knowledge backgrounds play an important role in the shaping and outcomes of the control activities (Shackleton et al. 2019a).

### Objectives

This paper describes and analyzes the outcomes of a 4-year *V. velutina* nest removal program (2016–2019) implemented by a municipality in Galicia (NW Spain), one of the European areas where the species is already well established. The removal of nests of *V. velutina* might be considered a partially faulty community-based or participatory management (Hill et al. 2020), where the knowledge and motivations of local community members on the species differ from those of practitioners, i.e., the individuals responsible for day-to-day management decisions and activities (Cook et al. 2014). The analysis of this program in a highly invaded locality allows us to finely explore non-standardized management practices at the local scale by using a set of cross-stakeholder data, and connect it to drivers such as risk perception and budget allocation. These drivers are of high interest for territories in the front of the invasion or where the species is considered already of concern due to its impacts elsewhere (e.g., Invasive Species Council and Monash University 2020).

In particular, we:

1. Describe and analyze the nest removal plan and practices in the municipality of Oleiros, where the personnel of the Emergency and Civil Protection Unit (ECP-Oleiros) are responsible for all the management practices concerning *V. velutina*. The analysis includes the temporal and spatial analysis of the distribution of the nests requested to be removed in relation to demographic factors (number and density of inhabitants of municipality administrative divisions).
2. Assess community engagement with the control of the species and identify drivers of citizens’ actions, including beliefs, risk perception and attitudes.
3. Analyze the interaction between the ECP-Oleiros personnel and the civilians by studying the problems and/or conflicts between them, the origin of those problems and potential solutions. This analysis includes a fine-scale estimation of nest removal costs as a potential factor of pressure on the ECP-Oleiros unit.

Through these three actions, we will argue that the analysis and study of the main factors that influence the perceptions of the citizens, as well as their prior knowledge of the problems associated with *V. velutina*, could facilitate the detection of possible barriers in the establishment of effective management plans, as well as the integration of the community within them. This could make it easier to reduce conflict, improve stakeholder participation and engagement and gather support for the measures envisaged in the management plans (Shackleton et al. 2019a).

## Material and Methods

### Area of Study

**Fig. 1:**
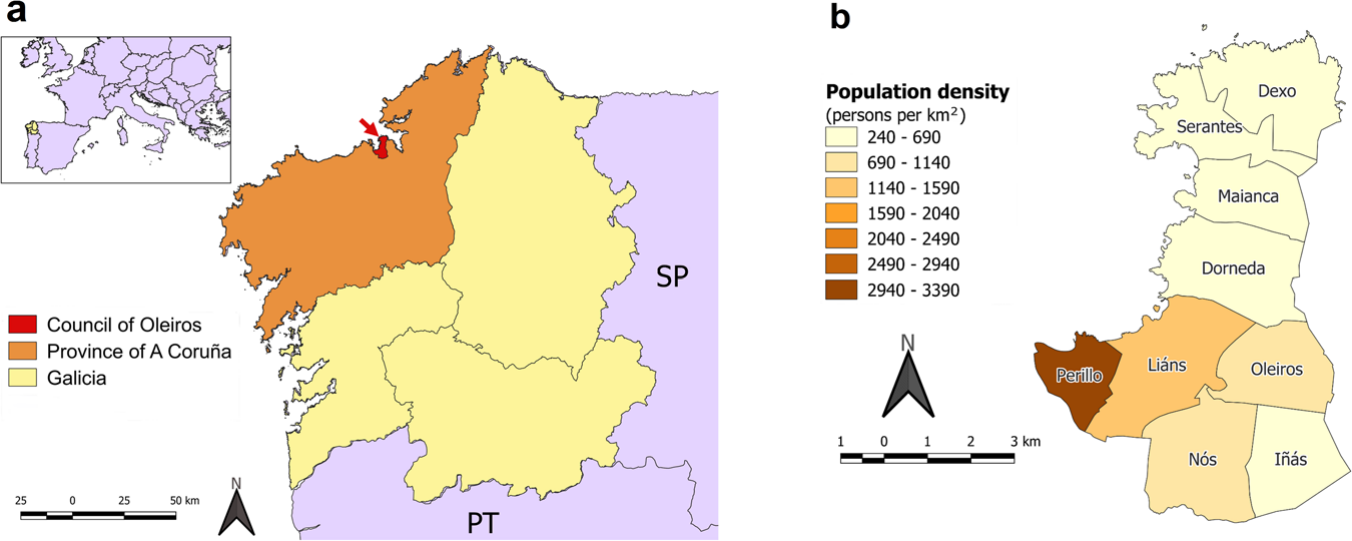
**a** Location of the study area in the autonomous region of Galicia in NW Spain (SP: Spain, PT: Portugal). **b** Territorial division and population density of the municipality of Oleiros

Our study was carried out in the municipality of Oleiros, which is located in the NW coast of the autonomous region of Galicia (NW Spain: 29 T 550882, 4798004) (Fig. 1). It covers an area of 44.28 km^2^ and a population of 36075 inhabitants (Instituto Nacional de Estadística 2019). It is a residential area where ca. 40% of the land cover is urban surface, which is mostly concentrated in the west coast close to the city of A Coruña (Fig. 1). The rest of the land is dedicated to agriculture (heterogeneous agricultural areas) and forests (coniferous forests, mixed forests and shrublands) (SIOSE 2014). The territory is divided administratively into nine parishes (Fig. 1), which are territorial entities below the municipality officially recognized by the Galician administration.

The parishes show a mean area of 4.92 km^2^ (min: 2.56 km^2^, max: 6.63 km^2^) and a population density that ranges from 240 to 3390 persons per km^2^ (Fig. 1). Following the classification of the degree of urbanization of Galician parishes and municipalities (Instituto Galego de Estatística 2016), the only parish that can be classified as a totally urban area is Perillo (Fig. 1), which is a dormitory town of the nearby council of A Coruña, while the rest of the parishes are constituted by suburbs and rural areas. Because of the clear differences in land use and demographic characteristics that might introduce confounding effects, Perillo will be excluded from all statistical analyses.

### Methodology

#### Nest data collection

Citizens’ requests for the removal of *V. velutina* nests in Oleiros started in 2014. Until January 2020 this task had been performed exclusively by the ECP-Oleiros unit, which is headed by a coordinator and has a total of 9 professional disaster-trained members. They recorded and destroyed, when technically possible, all the nests requested to be removed. This allowed us to calculate an annual rate of “Removal efficiency” (%) as the ratio of removed nests to requested nests (N.nests.removed*100/Total.nests.requested), where large values (close to 100%) mean high efficiency (see Table 1). Destruction could require extra visits of the ECP-Oleiros unit in order to check nest locations, to take decisions on the equipment to be used or to keep petitioners informed. Therefore, all nest destruction-related visits are considered as “Hornet activities”, and we define “Activity efficiency” as the ratio of all hornet activities to the number of requested nests (N hornet.activities/Total.nests.requested). Since each requested nest involves one hornet activity at least, the activity efficiency takes values larger or equal to one, and small values (close to 1) mean high efficiency (see Table 1).

**Table 1:** ECP-Oleiros nest removal activities

Year	Hornet activities	Nests			Nest density (*n*/km^2^)	Removal efficiency (%)	Activity efficiency
		Removed	Not removed	Total			
2016	1067	454	59	513	11.6	88.5	2.1
2017	1059	699	58	757	17.1	92.3	1.4
2018	840	631	53	684	15.4	92.3	1.2
2019	656	450	86	536	12.1	84	1.2
Total	3622	2234	256	2490			

Hornet activities: nest removal activities plus any extra travel to check nest location, inform citizens, etc. Removal efficiency: Removed*100/Total nests; Activity efficiency: Hornet activities/Total nests

Nest removal requests were recorded in a database provided by the regional administration, but for the sake of a better internal organization the ECP-Oleiros unit created an independent database by mid-2015, which was properly refined and used from 2016 to present. ECP-Oleiros nest removal database includes information on the location (parish), date of removal, staff that participated in the action and further observations such as the size of the nest (embryonic/mature nest), materials and techniques used, success in removal and any other information of interest.

Besides the nest removal database, ECP-Oleiros keeps a general detailed record of all the annual activities, time invested in each, material used, etc. that is published online (Concello de Oleiros 2021a). These reports were used to obtain information on the number of total activities dedicated to *V. velutina* control as well as time invested in those activities from 2016 to 2019.

#### Citizen Survey

Since nest removal is performed exclusively on request, we explored the perceptions or attitudes of the citizens facing the invasion of *V. velutina*, as well as beliefs or fears related to it. Because of this, we used a non-random sampling strategy, selecting attendants to the local Honey Festival in Oleiros as a community of interest for this work (Hill et al. 2020) in order to avoid answers of unaware or uninterested public.

A total of 173 surveys were conducted in November 2019, and interviewees were asked on the following topics (Online resource 1):

1. Whether they knew if they were allergic to bee or wasp stings.
2. The reasons for calling the emergency services in case they detected hornets or nests.
3. The motivation for getting involved in control actions and the methodologies selected for participating (e.g., installation of traps).
4. Whether they felt well informed or not on *V. velutina*.
5. Their opinion on the activities carried out by the local administration, which may indicate the degree of communication between both stakeholder groups.

The questions were mostly multiple-choice or open-ended, so choices selected for a question may exceed the number of respondents, causing percentages to add up to more than 100%.

#### Interviews with ECP-Oleiros Unit Personnel

By conducting individual interviews with ECP-Oleiros staff in November 2019 (Online resource 2), we tried to detect the most common challenges and obstacles for the control of *V. velutina*, as well as changes in practices they have been introducing during these years. The interview included sections on: (1) ECP-Oleiros decisions and practices for nest removal; (2) economic aspects; (3) design and details of the nest removal database; (4) citizens’ calls or inquiries to ECP-Oleiros on *V. velutina* and (5) other control activities.

Interviews were semi-structured and the interviewer was able to add questions that were not on the original list. Questions about nest data management were answered by the employee in charge of this task, while questions on economic aspects were answered by the coordinator of the unit.

**Fig. 2:**
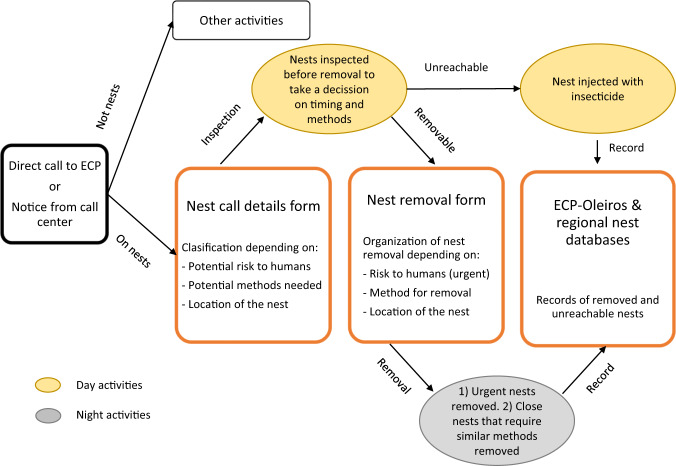
Decision-making diagram of nest removal activities elaborated after interviews to ECP-Oleiros staff and annual reports of the unit

Following ECP-Oleiros unit answers, a decision-making diagram on nest removal was created (Fig. 2).

These interviews were used also to assess the interaction and transmission of information between the ECP-Oleiros personnel and the local population by comparing responses on the same topic.

### Data Analysis

The number of annual nest requests were compared over the years using a Friedman test. To identify the significant differences between years, multiple pairwise Wilcoxon tests were performed using the Holm-Bonferroni correction method (Holm 1979). The relationship between the number of nest removal requests and demographic characteristics of the parishes such as number of inhabitants, area and population density, was studied with the Spearman correlation coefficient and using linear regression fits. The linearity and homocedasticity assumptions were not met in the linear fit, so the quadratic fit that fulfills all the required assumptions is also shown for visualization. Statistical significance was set at *p* < 0.05. All statistical analyses were performed using the R statistical software package (v. 4.0.3, R Development Core Team 2020).

We conducted descriptive analyses of the responses obtained from the survey to the Honey Festival participants, as well as sociological discourse analysis (Feindt and Oels 2005) to the responses obtained from interviews with the ECP-Oleiros crew. The results allowed us to diagnose the attitudes among local civilians towards *V. velutina*. We used pre-defined categories (service, training, efficiency, equitability, materials, budget) in our discourse analysis to identify and group the main issues of concern with our informants and to describe the interaction between practitioners and local civilians.

## Results

### Nest Removal Activities

**Fig. 3:**
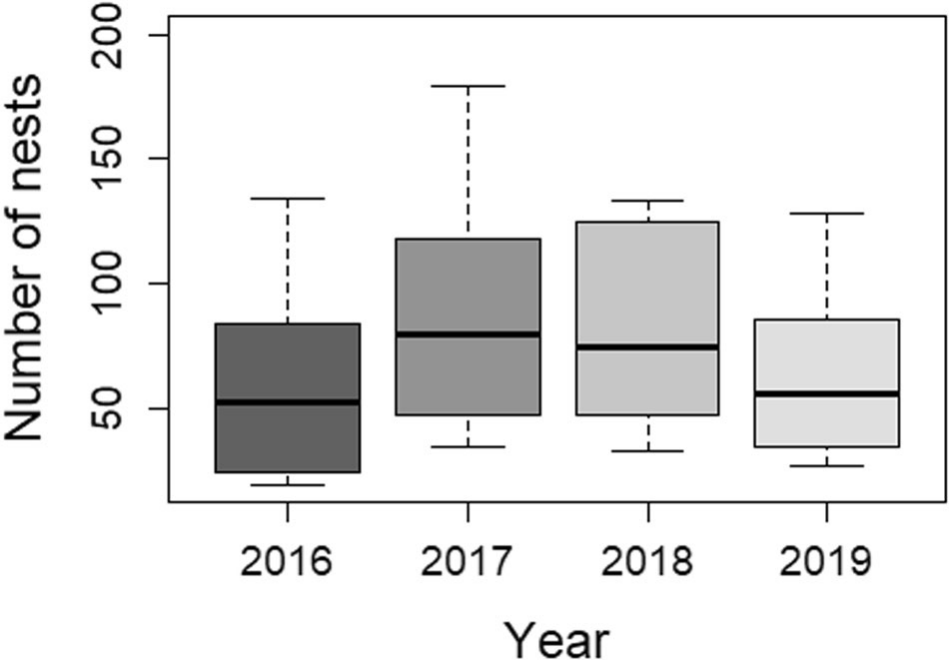
Number of nests requested to be removed (including both reachable and unreachable nets) recorded in the municipality in 2016–2019

**Fig. 4:**
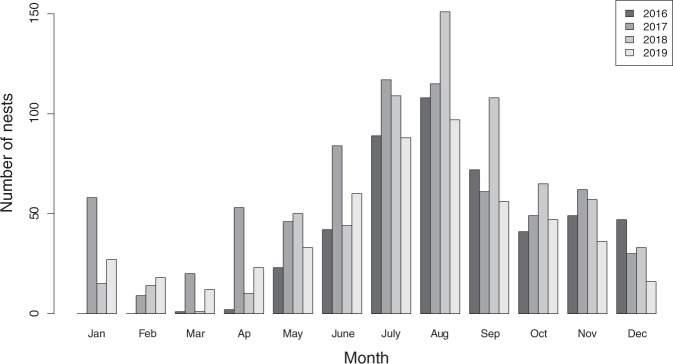
Monthly number of nests requested to be removed recorded in the municipality in 2016–2019

Annual reports show that the ECP-Oleiros unit has been dedicating most of their duties to nest removal activities for the last years (over 60% of the total number of activities in 2017 and 2018) (Concello de Oleiros 2021a), with a total of 2490 nest removal requests recorded from 2016 to 2019 (Table 1). Nest removal requests varied significantly among years (*p* = 0.0037), with significant differences between 2016–2017 (*p* = 0.047), 2017–2019 (*p* = 0.047) and 2018–2019 (*p* = 0.047) (Fig. 3), and a clear peak in summer months (Fig. 4).

Interestingly, the number of activities dedicated to hornets (i.e., nest removal plus any previous or subsequent travel or activity in the nest location) doubled the total number of nest removal requests recorded in 2016, while this ratio descended notably in 2018 and 2019 (see “Activity efficiency” in Table 1). We detected also an increase in the number of non-removed nests registered in 2019 (Table 1).

**Fig. 5:**
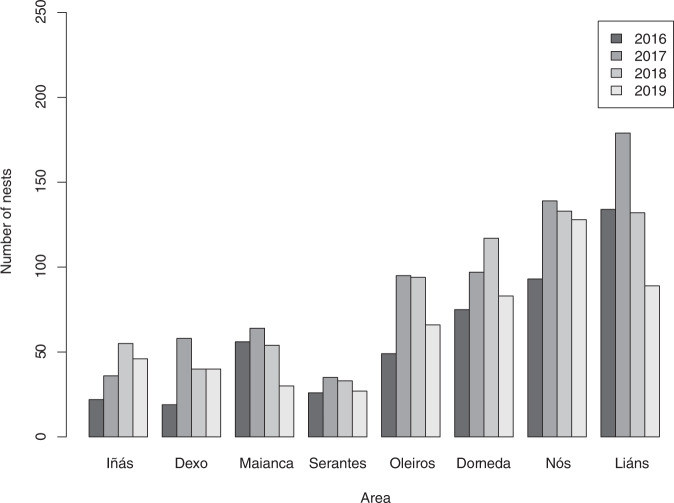
Number of nests requested to be removed in Oleiros parishes (in increasing order of population) in 2016–2019

**Fig. 6:**
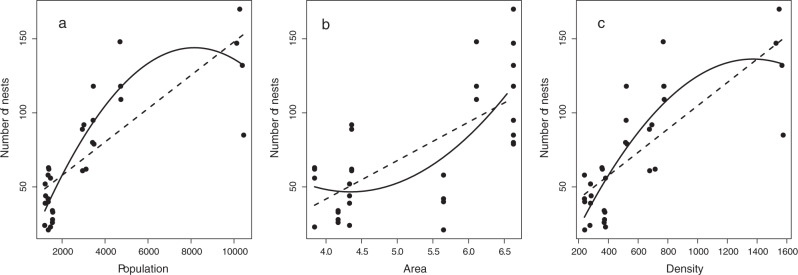
Number of nests requested to be removed in the municipality depending on the number of inhabitants (**a**), the area (**b**) and population density (**c**). Predicted values with a linear regression fit (dashed lines) and quadratic fit (solid lines)

As for the territorial divisions into the municipality, the number of nest removal requests was higher in the parishes with higher number of inhabitants (Fig. 5). The number of requests was significantly correlated with the number of inhabitants (*r* = 0.787, *p* < 0.0001), the area (*r* = 0.699, *p* < 0.0001) and the population density (*r* = 0.773, *p* < 0.0001) (see Fig. 6 for visualization).

### Community Engagement in the Management of *V. velutina*

Only 2.9% of the interviewed Honey Festival attendants declared that they are allergic to stings. However, as shown in Table 2, most people (69.8%) would call the emergency services because of the presence of a nest, while 15.7% of the respondents would call because of the presence of either a nest or a hornet. Only 0.6% would call because of the mere presence of hornets. The exact question posed to the respondents was “Would you call emergency services in case you detect hornets? And in case you detect a nest? Why?” (see Online resource 1). This factor is important, as calls made for the mere presence of hornets are normally useless, not conducting to the detection and removal of nests, and fear to the insect is usually the reason behind such calls. Indeed, the most important reason of the public to call emergency services in case of nest location is its importance for management (75%), which indicates a remarkable degree of involvement in the management of the invasion.

**Table 2:** Reason for calling emergency services of the Oleiros Honey Festival public (a) and motivation and methodologies for participating in control actions (b)

	Answer	Reason or method	%
a) Call to emergency services	Call for nest (69.8%)	Management	75.0
		Fear	23.3
		Other	1.7
	Call for nest or hornet (15.7%)	Management	22.2
		Fear	63.0
		Disinformation	7.4
		Other	7.4
	Do not call (14%)	Unconcern	55.2
		Self-management	37.5
		Other	77.3
	Call for hornet (0.6%)	Fear	100.0
b) Participation in control	No (63.4%)	No opportunity or know-how	95.6
		Unconcern	16.7
		Fear	19.4
	Yes (36.6%)	Management	76.4
		Fear	28.6
		Economical reasons	33.3
		Traps	63.5
		Nest removal	42.9

Most of the public (63.4%) do not actively participate in control activities because they consider they do not have the opportunity or the know-how (unconcern or fear are less frequent reasons) (Table 2). Trapping is the most common method (63.5%), while nest removal is less frequent. The survey also shows that 55.4% of the participants felt well informed on the species, and TV, web resources and social networks were the most common media used for obtaining information (Table 3).

**Table 3:** Perception of the public on their degree of information on *V. velutina* and media selected for getting information

Answer	Media	%
Sufficiently informed (55.4%)	TV	33.7
	Web & social media	45.7
	Dissemination	28.3
	Press	40.2
	Local knowledge	14.1
Insufficiently informed (44.6%)	TV	50
	Web & social media	33.8
	Dissemination	17.6
	Press	35.1
	Local knowledge	21.6

In general, interviewees are skeptical on the ability of administrations to manage the invasion and do not have a good opinion of their management activities. They ask for organizational improvements in the management program (40.6%) and more resources (14.5%), as well as more research and information by the administrations (23.9%). An important percentage did not answer this question (21%), indicating that they are not aware of the activities carried out by the administration.

### Practitioners’ Management Practices and Interaction with the Local Population

ECP-Oleiros annual reports and their database provided valuable information on management practices, that was summed up in Fig. 2. Interviews to ECP-Oleiros staff allowed us to better understand decisions and practices for nest removal. Among the improvements made by the staff during the period analyzed in this work (2016–2019), they highlighted:

1. Service to citizens. Any call on the species was answered, even in doubtful cases (misidentifications were common in early years). Visits to places and claimants were considered to be important in order to calm the citizens, and they did so even in cases of nests already considered inaccessible in previous visits. The staff admitted notable improvements over the years in their knowledge of the species and their ability to communicate with citizens.
2. Protocols. The initial training of the unit on removal methods was provided by beekeepers through informal collaborations. The staff participated in formal courses, though they considered these courses to be scheduled late, and they missed clear guidelines and methodological protocols. They considered that field experience was their main way to improve their labor, and used scattered web resources and social media for solving doubts. Interestingly, they found press news to be rarely informative and to increase the feeling of danger of the species.
3. Efficiency. One of their main aims was to reduce traveling, as this was time-consuming and restricted the availability of personnel, material and vehicles for other activities and emergencies. Thus, it was crucial to improve the quality of the information obtained from direct calls to the ECP-Oleiros headquarters concerning correct identification of the species, location, nest characteristics, material that might be needed, etc. Another objective was to reduce the removal of nests already abandoned and damaged during winter, so they improved the information given to people by explaining that nests are not reused.
4. Equitability. The activities were very frequent at certain periods of the year and required a notable effort, as most nests were removed during the night. Consequently, they implemented an internal organization system to make work equitable and avoid conflicts in relation to responsibilities to remove problematic nests (e.g., those in high trees or intricate locations), responsibilities to organize material, update databases, etc.
5. Budget. Purchase of personal protective equipment and adequate materials was an important problem at the beginning of the invasion, as a wide array of products were offered to the unit by sales representatives without adequate testing evidence or guidance support. This resulted in some superfluous purchases and required an extra effort of investigation by the coordinator of the unit in order to find adequate material at a fair price. The correct use of equipment and materials, including chemical products such as pesticides, required the implementation of internal protocols.

Since nest removal is free of charge for citizens, we tried to estimate a realistic value of the costs of this activity to the Council by taking into account: distance from the ECP-Oleiros headquarters to place of removal, total time invested in removing a nest depending on its size (embryonic or mature nests) and costs of personnel and material (consumables and time of use of inventorial equipment). Estimates were computed by the coordinator of the unit by referring to a public document of the council on costs of emergency material and personnel (Concello de Oleiros 2021b) through self-experience on the activity. Estimated costs depending on nest characteristics and material used ranged from 40–200 € approximately (Table 4). This is roughly aligned with the mean costs per nest in the whole region of Galicia since 2020 (73€ in 2020 and 62.5€ in 2021; Xunta de Galicia, unpublished data), although removal of mature nests in our study were estimated to be always over 100€ (Table 4). Thus, for a mean number of about 560 removed nests per year (see Table 1) by using the telescopic pole method (the most common; see Table 4), and assuming that half of them were embryonic, the Council of Oleiros may have spent from 60500 to 85800 € yearly in the studied period, not including in the calculation travels and treatments of unreachable nests.

**Table 4:** Examples of combinations of characteristics that influence the cost of nest removal

Cost	Nest type	Access	Method	Time	Distance	Cost (€)
< 100€	Embryonic	Easy	Manual	Day	Short	37.2
		Easy	**Pole**	**Day**	Short	57.6
		Easy	Fire ladder	Night	Short	84.9
>100€	Mature	Difficult	Fire ladder	Night	Long	102.4
		Easy	Pole	Day	Long	114.9
		Difficult	Manual	Night	Short	170.5
		Easy	**Pole**	**Night**	Short	202.5
		Difficult	Pole	Night	Long	227.3

Most frequent method and time of removal in bold

Most of the surveyed public of the Honey Festival (86%) indicated that they would call emergencies for the presence of a nest or even hornets, which coincides with the experience of the ECP-Oleiros unit. However, while few of them declared to be allergic and they indicated no major fear or feeling of danger to the species, ECP-Oleiros staff highlighted that the most common arguments for nest removal requests were allergies or other health-risk conditions. As for information about the species, the participants of the Honey Festival taking part in the survey considered themselves to be well informed. However, ECP-Oleiros staff indicated that they frequently had to answer questions on identification, nest construction behavior, aggressiveness of the species, or the use of traps. Indeed, the staff recommended citizens to use traps for reducing the presence of hornets in their gardens, but never recommended unprofessional nest removal.

An interesting remark made by the ECP-Oleiros unit staff was that they felt that even if concern for the species seems to be decreasing in recent years, citizens requesting nest removal use the motives of fear and danger related to allergies to urge the staff, which obliges them to attend both the content and relational dimensions in communication to avoid conflicts.

## Discussion

### Nest Removal Requests and Characteristics of the Territory

Nest densities reported in this work are among the highest reported in other areas invaded by *V. velutina* (e.g., Italy: Bertolino et al. 2016; France: Monceau and Thiéry 2017; Portugal: Carvalho et al. 2020). Because nest removal records depend on citizens’ requests, territorial characteristics such as local population density are expected to affect them (Choi et al. 2019; Robinet et al. 2017). However, subtler characteristics that influence detection (e.g., type and density of vegetation, landscape features, etc.) and also the social perception of the species might also have an impact on such numbers (Choi et al. 2019; Franklin et al. 2017). In addition, management strategies adopted by administrations and news disseminated by media (Do et al. 2019) might have an influence on citizens’ willingness to request nest removal (Hester and Cacho 2017).

Landscape in Oleiros municipality offers a potential high carrying capacity for the species with a predominance of both artificial surfaces and agricultural areas, which are suitable habitats for *V. velutina* (Villemant et al. 2011), and facilitates nest settlement. As for social aspects that might influence removal requests, the Council of Oleiros has enacted and made public regulatory policies against invasive species. Thus, characteristics of both the territory and the community might have contributed to boost the number of recorded nests in the municipality. However, potential explanations to variations detected in the period of study are not straightforward, as they might be the consequence of multiple social factors (e.g., in 2018 three deaths by sting happened in the same week in the region and were reported by national media) or climate-dependent natural variations (Choi et al. 2019).

### Drivers of Practitioners’ Management Practices

There are currently no standardized control methods for *V. velutina* that would allow a coordinated response in different countries (Leza et al. 2021). However, recent reviews (Choi et al. 2019; Turchi and Derijard 2018) and management strategies derived from projects (Porporato et al. 2019) describe the diversity of current practices. In many cases, communication issues among stakeholders, in particular between scientists and beekeepers, have been detected (Requier et al. 2020). Similar issues are highlighted by the ECP-Oleiros staff during the interviews, who missed extra expert guidance and more knowledge transfer coming from scientists and the government.

The main drivers of practitioners’ decision-making seem to reflect well the spirit of a public service (removal of every nest, visits to claimants, efficient phone communication), the search for optimal internal functioning (training, equitability in task division, proper use of equipment) and cost-effective responsibility as a municipal department (efficient use of time and resources). These attributes make practitioners’ activities adequate for the correct collection of citizens’ data in the passive surveillance of *V. velutina* nests (Hester and Cacho 2017). However, every single nest was eliminated regardless of the economic costs, which indicates that their nest removal strategy during this period might be described as if the invasion was a “problem to solve once”, instead of a problem that is here to stay and must be managed in the long term (Cottet et al. 2020).

### The Role of Budget in Nest Removal Programs

A debate that deserves attention is the economic sustainability of management activities (Larson et al. 2011). Nest destruction is expensive, and costs have been estimated to amount to €23 million between 2006 and 2015 in France (Barbet-Massin et al. 2020). These authors extrapolated them for *V. velutina* climate suitable areas based on costs of ten French administrations that subsidize this activity, and they expect expenses to increase as the species invades new areas. As for Spain, *V. velutina* has been already included in the list of the ten IAS that generate the greatest expense in management (Angulo et al. 2021).

Reinforcing nest removal has been suggested to be a suitable strategy for limiting the spread and density of *V. velutina*. However, as stated by Robinet et al. (2017), effects of control efforts remain unclear, and a lack of reports on successful cases may be the cause of the citizens’ skepticism about the ability of administrations to manage the invasion. Our analysis contributes to the discussion about the optimal resource allocation for nest removal activities, which may differ depending on the goal to achieve. If the objective is the eradication of the species in newly or scarcely invaded areas, it would be desirable to eliminate all nests at any time of the year, particularly before males and gynes begin to emerge (Monceau et al. 2014; Porporato et al. 2019). Managers should secure funding for the completion of these projects, including post-elimination surveillance (Leza et al. 2021). However, in cases where the final goal of nest removal is to limit the spread of the invasion or to reduce the density of *V. velutina*, a detailed cost-benefit analysis should be made in order to produce guidelines for optimal removal practices in time and space. Ideally, the analysis should include both direct expenses of nest removal activities and benefits and costs derived from the environmental impact and losses generated by the species (Diagne et al. 2021; Larson et al. 2011). Yet, at present, there is still little evidence available for *V. velutina*, though Robinet et al. (2019) estimated a reduction in the spread of the invasion depending on the percentage of nests removed, and Requier et al. (2020) estimated the impact on beekeeping depending on nest densities.

A more contentious element in this debate is the need to protect civilians because of the potential fatalities caused by stings, and whether this task should be publicly funded by administrations and free of charge for citizens, as in the present case, or just subsidized (e.g., Barbet-Massin et al. 2020). Choi et al. (2019) have already noted important drawbacks of the free-of-charge strategy in South Korea. They indicated that emergency teams removing low-threat nests were diverted from attending other emergency services, and the strategy failed to reduce the threat of the species perceived by the citizens. Thus, socio-political factors might play a role on the willingness of administrations to invest in nest removal and content social demands, whether reasonable or disproportionate.

### Public Views and Interaction with Practitioners

Even if practitioners’ management practices are adequate and adjust to recommended guidelines, control of invasive species can be highly controversial. Public perceptions on IAS play a key role in the promotion or obstruction of effective management, and these perceptions depend on key factors such as species traits, species effects or people’s environmental knowledge and beliefs around the species (Shackleton et al. 2019b). Hymenopterans are generally perceived as dangerous insects, but charismatic species such as honeybees are valued because of their role in pollination. Indeed, awareness of the role of pollinators and their decline is already widespread among laypeople and stakeholders (Hevia et al. 2021), and local knowledge of pollinators is being explored as a source of information for conservation actions (Burns et al. 2021). All these factors might contribute to the negative reputation of *V. velutina* in invaded areas and the anxiety behind nest removal requests, as predation on honeybees as well as human fatalities caused by stings are among the topics that have deserved attention both in scientific literature and mass media (Do et al. 2019). It is important to highlight that beekeepers, researchers and policy makers have been identified as important stakeholders in *V. velutina* management (Carvalho et al. 2020; Requier et al. 2020). However, the health issues connected to the species and the predominance of nest removal as a control technique make local population also an important stakeholder whose feelings, views and opinions should be taken into consideration (Courchamp et al. 2017; Hester and Cacho 2017).

The implementation of a closer dialog among stakeholders might help to get to an agreement on whether resources to emergencies should be invested primarily to control an already established invader, as in the present case, or if such resources should be directed to make an efficient reduction of their socioeconomic, health and ecological impact. This requires a clear exposition of methods and results by managers, practitioners and/or facilitators to the public, including the economic and organizational aspects. In the particular case of *V. velutina*, the contribution of the public in the co-production of knowledge should be favored, because they can offer valuable information on the results and consequences of management practices. Expert knowledge transfer should also be fostered, in the form, for example, of expert-based environmental education programs to train citizens on the ecology of the species so that their interaction with practitioners is less driven by unfounded fears and more by state of the art scientific knowledge (e.g., on the state of the invasion or the defensive behavior of the species).

## Concluding Remarks

The information gained from the present diagnosis, together with the analysis of practices and social aspects related to the management of *V. velutina*, might be framed into an adaptive management strategy, and our results might be incorporated to optimize management plans in an iterative process. This is of great importance at the present state of the invasion in some European areas where *V. velutina* is a recent IAS or where models predict a potential future invasion, but also for areas where management has not been evaluated and discussed in depth.

The data provided in this paper support the crucial importance of environmental education programs that seek to increase the knowledge of the general public about the economic and health threats of IAS, as they could reduce the pressure on practitioners so that they can be more efficient. This would require enhancing communication and promoting social learning between experts and local population, either through targeted actions such as information points or meetings with affected communities or through non-targeted actions such as information campaigns in media and social media. This will contribute to control misinformation, irrational beliefs and fake news or sensationalist and misleading headlines, and to ease the relation between administration and local people.

## Supplementary information

Online Resource 1

Online Resource 2

## Data Availability

Data used in this work come from public sources and are available under request.
