# Supplementary material for: Of Fears and Budgets: Strategies of Control in Vespa velutina Invasion and Lessons for Best Management Practices
Source: Environ Manage. 2022 Jul 28;70(4):605–17. doi: 10.1007/s00267-022-01690-z (PMC9439987; doi:10.1007/s00267-022-01690-z)
Supplement: Supplementary file 1 — Online Resource 1 [file 267_2022_1690_MOESM1_ESM.pdf]

Of fears and budgets: Strategies of control in *Vespa velutina* invasion and lessons for best management practices

Environmental Management

Tamara Pazos, Patricia Álvarez-Figueiró, Jose A. Cortes-Vazquez, María Amalia Jácome, **María J. Servia**

Dep. of Biology. Faculty of Science. University of A Coruña, UDC. Campus da Zapateira s/n, 15071 A Coruña, Spain. **maria.servia@udc.es**

Outline of the questionnaire used to obtain information from public attending to the Oleiros Honey Festival - November 2019

Basic data of the interviewed person

1. Gender (M/F):
2. Age:
3. Are you allergic to *V. velutina* stings?
   IF SO: How do you know or why do you think you are allergic?

Motivation to call emergency services

4. Would you call emergency services in case you detect hornets? And in case you detect a nest? Why?

Participation in control actions

5. Have you ever participated in activities for the control of *V. velutina*?

   a) IF SO: How did you participate? Options:

      a.1) Installation of traps

      a.2) Removal of nests

      a.3) Other:

   b) IF SO: Why did you participate?

b.1) As a contribution to control the expansion of *V. velutina*

b.2) It has an impact in my economy (beekeeping, agriculture…)

b.3) I am afraid of hornets

b.4) Other:

c) IF NOT: Why did you not participate?

c.1) I did not have the opportunity

c.2) I do not have the know-how

c.3) I am afraid of hornets

c.4) It is not my responsibility

c.5) Other:

Information on the species

6. Do you feel you are well informed on *V. velutina*?

a) Where do you get information on the species?

a.1) Local newspapers

a.2) Scientific papers

a.3) TV

a.4) Social media (Facebook, Twitter…)

a.5) Other:

Role of administrations

7. Do you think the Council or the regional administration are correctly managing the invasion?

Comments:
