# Supplementary material for: Of Fears and Budgets: Strategies of Control in Vespa velutina Invasion and Lessons for Best Management Practices
Source: Environ Manage. 2022 Jul 28;70(4):605–17. doi: 10.1007/s00267-022-01690-z (PMC9439987; doi:10.1007/s00267-022-01690-z)
Supplement: Supplementary file 2 — Online Resource 2 [file 267_2022_1690_MOESM2_ESM.pdf]

Of fears and budgets: Strategies of control in *Vespa velutina* invasion and lessons for best management practices

Environmental Management

Tamara Pazos, Patricia Álvarez-Figueiró, Jose A. Cortes-Vazquez, María Amalia Jácome, María J. Servia

Dep. of Biology. Faculty of Science. University of A Coruña, UDC. Campus da Zapateira s/n, 15071 A Coruña, Spain. maria.servia@udc.es

Outline of the questionnaire used for interviewing staff of the Emergency and Civil Protection Unit (ECP-Oleiros) - November 2019

BLOCK 1. ECP-Oleiros activities and practices in nest removal

1.1 Do you remove nests at a specific season of the year or all year round?

a) IF SO... why are they not removed at certain seasons?

1.2 Do you prioritize any case among all nest removal demands?

a) Which cases are prioritized and why?

1.3 Which is the most commonly used method for nest removal and why?

1.4 How did you implement the techniques you use at present for nest removal (bibliography, testing...)?

1.5 Are you up to date on new techniques and / or devices used for nest removal or neutralization? (IF SO… give examples)

1.6 Do you try to remove or neutralize a nest even when it seems difficult?

a) In which cases do you desist from removing or neutralizing a nest?

BLOCK 2. Economic aspects of nest removal. *Cost estimation of activities answered by the coordinator of the ECP-OLEIROS and the person in charge of the "V. velutina service"*

2.1 How long does it take to remove/neutralize a nest (travel time excluded)?

2.2 Which consumable material (gloves, insecticide, etc.) do you need?

a) IF SO… which is the average lifespan of each and how much do they cost?

2.3 Reports show that telescopic pole and ladder truck are the most commonly used materials for nest removal. Are there any additional costs for the use of these devices?

2.4 Which type of vehicle do you normally use for nest removal activities (visits and neutralization/removal?

2.5 Do you use any high-altitude rescue equipment for high nests?

BLOCK 3. ECP-Oleiros nest-removal database. *Questions on details for data curation not included.*

3.1 For nests recorded in 2016-early 2017 you indicate whether it is a primary or secondary nest.

a) Why did you stop recording this information?

c) Do you think this information might be useful?

d) IF SO… should this information be added again?

3.2 In some cases, the location of the nest is indicated in detail (i.e.: "At the top of the front door", "On a retaining wall", "In an apiary", etc.).

a) Why do you indicate the location only in some cases?

b) Do you think this information is necessary before or after the removal?

c) IF SO… why?

3.3 You register the method used to remove the nests (telescopic pole, ladder truck…) only in certain cases, these cases being less numerous after 2017.

a) Why is it only specified in some cases?

b) Why has this type of annotation changed, being less frequent after 2017?

c) Which method did you use in those cases where the removal method is not specified?

d) Do you think it is useful to know, and therefore add, the type of method used in each case?

e) Why / why not?

3.4 After 2017 you highlight the term "NEUTRALIZED" for some nests (yellow color).

a) Why is it necessary to highlight these nests?

3.5 Regarding inaccessible nests, we observed that the reason for non-removal is more detailed in some cases than in others (i.e.: "Not accessible" vs. "Private area without clearing").

a) Is there a reason for this difference?

b) Do some cases need to be more detailed than others?

c) IF SO… which ones and why?

3.6 Do you think it is necessary to specify the main reason for non-removal (i.e.: "It is above a river", "It is very high", "Lack of adequate material")?

a) IF SO… why?

3.7 Which is the most important "observation" to consider for removed and unremoved nests? Why?

BLOCK 4. Calls / queries from the local population

4.1 Which are the most common reasons for people to demand the removal of nests (e.g. nest location, allergies, contribution to management, etc.)?

a) Are these reasons recorded?

4.2 Is there any reason for nest removal demands that has become more/less common?

a) IF SO... which one and why do you think it has become more/less common?

4.3 Do you think you are able to solve any question concerning *V. velutina* (identification of individuals and nests, general information about its life cycle, allergic reactions, etc.)?

4.4 Which is the most frequent type of question (except on the removal of nests)?

4.5 What kind of questions and demands are the most difficult to solve and why?

4.6 What do you do when you do not know how to solve the question?

4.7 Do you use any particular resource (i.e., an image database, a web resource…) to look for information?

a) Would it be useful to you to have access to a FAQ content type to answer questions?

4.8 How do you feel when transmitting information to the population (confident, insecure on the use of technical vocabulary…)?

a) Do you think people understand your explanations?

4.9 Do you consider that you are well formed on aspects that can influence the management of *V. velutina* (identification, biology, behavior, allergies…)?

a) What aspect or aspects are considered a priority?

BLOCK 5. Other control activities

5.1 Apart from nest removal/neutralization, can you think on any other activity that might be useful for the control of *V. velutina*?

5.2 Do you set traps at any season of the year?

a) Do you consider trapping as a useful method?

b) Would you recommend this activity to the local population?

5.3 Do you think the local population should carry out any specific management activity?

a) IF SO… which ones and why?

5.4 What are the activities, tasks, or tips that local population don't want to perform, although you recommend it?

a) IF SO… why do you think they do not?

5.5 The Xunta de Galicia has announced a change in the management of the removal of nests that would release you from this work. What do you expect to happen in the coming years?
